# Netrin Signaling Breaks the Equivalence between Two Identified Zebrafish Motoneurons Revealing a New Role of Intermediate Targets

**DOI:** 10.1371/journal.pone.0025841

**Published:** 2011-10-07

**Authors:** Laura A. Hale, Daniel K. Fowler, Judith S. Eisen

**Affiliations:** Institute of Neuroscience, University of Oregon, Eugene, Oregon, United States of America; Pomona College, United States of America

## Abstract

**Background:**

We previously showed that equivalence between two identified zebrafish motoneurons is broken by interactions with identified muscle fibers that act as an intermediate target for the axons of these motoneurons. Here we investigate the molecular basis of the signaling interaction between the intermediate target and the motoneurons.

**Principal Findings:**

We provide evidence that Netrin 1a is an intermediate target-derived signal that causes two equivalent motoneurons to adopt distinct fates. We show that although these two motoneurons express the same Netrin receptors, their axons respond differently to Netrin 1a encountered at the intermediate target. Furthermore, we demonstrate that when Netrin 1a is knocked down, more distal intermediate targets that express other Netrins can also function to break equivalence between these motoneurons.

**Significance:**

Our results suggest a new role for intermediate targets in breaking neuronal equivalence. The data we present reveal that signals encountered during axon pathfinding can cause equivalent neurons to adopt distinct fates. Such signals may be key in diversifying a neuronal population and leading to correct circuit formation.

## Introduction

Developmentally equivalent cells adopt distinct fates in response to signals from within an equivalence group or from neighboring cells [1], [2]. The interactions that enable equivalent neurons to develop cell-specific axon trajectories are generally thought to occur early [3]. However, early interactions are unlikely to allow developmentally equivalent neurons to extend axons to a common target and then form distinct synaptic fields within that target [4]. This raises the possibility that signals encountered later in development may contribute to the formation of local circuits by breaking equivalence between neurons, thus diversifying the neuronal population [4]. Here we investigate the molecular identity of a signal encountered during axon pathway navigation that breaks equivalence between two motoneurons.

The molecular mechanisms by which developmentally equivalent neurons adopt distinct fates are not entirely clear. Neuronal equivalence has been defined by cellular studies showing that two neurons have the potential to develop a particular identity, but that interactions determine which cell realizes that potential by adopting a so-called preferred fate [5]. The other cell is then forced to adopt a non-preferred fate. In some cases the interactions may occur directly between the equivalent cells, suggesting that a single signal is sufficient. In other cases the interactions require not only the two equivalent cells, but also another cell type, suggesting that breaking neuronal equivalence in these cases involves at least two distinct signals [6].

**Figure 1:**
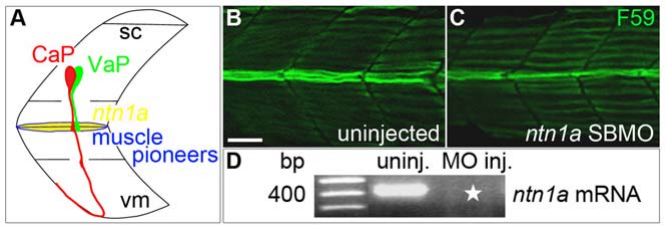
Ntn1a expressed in the muscle pioneers is a candidate signal for stopping VaP axon outgrowth. **A**. Schematized view of one spinal hemisegment including spinal cord (sc) and overlying muscle. CaP (red) and VaP (green) axons contact muscle pioneers (blue) at the first intermediate target. The VaP axon stalls at the muscle pioneers while the CaP axon continues into ventral myotome (vm). *ntn1a* mRNA (yellow) is expressed in muscle pioneers [16] . This image and following images are oriented laterally with rostral to the left. **B–C.** Projected confocal stacks of (**B**) uninjected and (**C**) *ntn1a* SBMO-injected embryos labeled with F59, a slow muscle fiber marker [70]. At 24 hpf, muscle pioneers (green fibers in the middle of the images) are present in both control and MO-injected embryos. **D.** RT-PCR results confirming uninjected embryos have wildtype *ntn1a* (450 bp band) that is absent from embryos injected with 5.6 ng of *ntn1a* SBMO (star). Multiple samples were run in this gel. Lanes containing samples unrelated to *ntn1a* were cropped from the figure. **Scale bar**  = 20 µm.

Zebrafish embryos possess two initially equivalent spinal motoneurons that later adopt distinct fates (Figure 1A) [7]. One of these motoneurons, CaP, is present in all spinal hemisegments and extends a long axon that innervates ventral muscle [8]. The other motoneuron, VaP, is present in only about half of the spinal hemisegments, extends a short axon, and typically dies during embryonic development [7], [9]. CaP and VaP are initially indistinguishable by morphological and molecular criteria. Their axons extend out of the spinal cord directly to an intermediate target, the muscle pioneers (Figure 1A). The muscle pioneers are a small set of identified muscle fibers that define the horizontal myoseptum separating dorsal and ventral myotome [10]. Both the CaP and VaP axons pause at this intermediate target. They then exhibit different behaviors that reveal that the cells have adopted distinct fates [6], [7]. One cell extends an axon beyond the muscle pioneers and this cell becomes CaP. In contrast, the axon of the other cell remains at the muscle pioneers and this cell becomes VaP [6], [7]. Despite these behavioral and morphological differences, to date no molecular markers that distinguish CaP and VaP at any stage of their development have been described. Here we refer to these cells as “CaP/VaP” at early stages when they are indistinguishable, and as “CaP and VaP pair” at later stages when they are morphologically distinct.

The cellular interactions that determine which CaP/VaP will become VaP are complex. A VaP develops only in a spinal hemisegment in which a CaP is also present; when there is only a single cell, it always develops as CaP [9]. However, this single cell has the potential to develop as VaP if it is transplanted next to a developing CaP [7]. When there are two CaP/VaPs, ablation of one cell of the pair before axogenesis causes the remaining cell to develop as CaP [7]. Ablation of CaP at later stages, after its axon has extended beyond the muscle pioneers, allows the adjacent VaP to develop into CaP [7]. In constrast, ablation of VaP has no effect on the adjacent CaP [2], [6]. Thus, CaP is the preferred fate of both CaP/VaPs. In addition, CaP must be present for the adjacent cell to adopt the VaP fate [6]. The muscle pioneers are also necessary for a CaP/VaP to adopt the VaP fate [6]. In the absence of the muscle pioneers, axons of both CaP/VaPs extend beyond the horizontal myoseptum intermediate target and the potential VaP essentially develops as a second CaP. Collectively, these data suggest that both muscle pioneer-derived and CaP-derived signals are necessary for VaP specification [6]. The molecular identities and timing of these two signals have yet to be determined.

In this paper we test the hypothesis that Netrin 1a (Ntn1a) is the muscle pioneer-derived signal that breaks equivalence between CaP and VaP. Netrins are a family of secreted proteins that can attract or repel axons (reviewed by [11]). Netrins also function in other capacities, including synaptogenesis, cell migration, cell survival, and tissue morphogenesis (reviewed by [12], [13], [14]). Netrins act through several receptors that can function independently or together, and thus mediate different Netrin responses by the same cell [14], [15]. Zebrafish *ntn1a* is expressed by the muscle pioneers (Figure 1A) around the time of CaP/VaP axon contact [16], making muscle pioneer-derived Ntn1a an excellent candidate for promoting VaP cell fate. Cell fate specification is not a function normally ascribed to axon pathfinding intermediate targets, thus it could represent an unrecognized mechanism involved in establishing neuronal circuitry.

## Results

### Netrin 1a is a muscle pioneer-derived signal necessary for VaP fate

**Figure 2:**
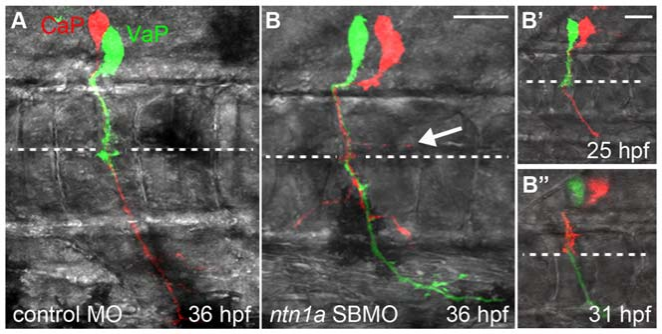
Ntn1a is necessary to prevent a second CaP/VaP axon from extending beyond the muscle pioneers. Projected confocal stacks of living dye-labeled motoneurons in one spinal hemisegment. **A.** VaP axon stalled at muscle pioneers (dashed line) at 36 hpf in standard control-MO injected embryo. **B.** *nt1a* SBMO injected embryos had two CaP/VaP axons that extended beyond the muscle pioneers. One CaP/VaP cell also extended an ectopic branch (arrow) along the horizontal myoseptum. **B'-B”.** The same cells as shown in B. At 25 hpf (**B'**) the red cell was a CaP, but by 31 hpf (**B”**) the red cell had retracted its axon to the muscle pioneers intermediate target and the green cell had become CaP. **Scale bars**  = 9 µm in A, B and 20 µm in B', B”.

**Figure 3:**
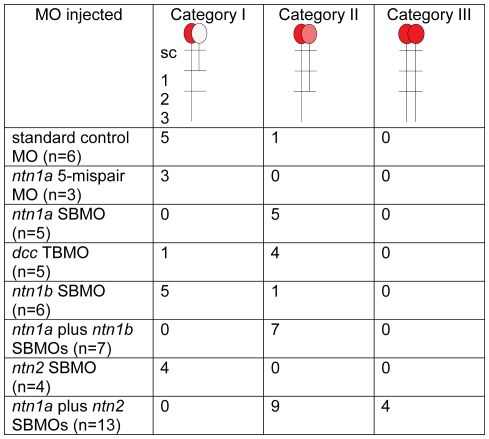
Netrin signaling prevents the VaP axon from extending into ventral muscle. We observed three categories of axon phenotypes in MO-injected embryos. In Category I, CaP axons extend into ventral muscle and VaP axons extend no farther than the muscle pioneers (1). In Category II, CaP axons extend into ventral muscle and a CaP-like axon extended beyond the muscle pioneers, but no farther than the level of the hypochord (2). In Category III, both CaP and VaP extend axons to the third intermediate target (3), thus both cells developed as CaPs.

We predicted that Ntn1a prevents one of the CaP/VaP axons from extending beyond the muscle pioneers, forcing that cell to acquire the VaP cell fate. To test this hypothesis, we knocked down Ntn1a function with a previously published splice-blocking (SB) morpholino antisense oligonucleotide (MO; Table 1). Similar to previously reported results [17] we did not observe changes in muscle pioneers in *ntn1a* SBMO-injected embryos (Figure 1B–D). To observe whether CaP and VaP were altered after knocking down Ntn1a we labeled individual CaPs and VaPs within the same hemisegment with different fluorescent dyes and followed their development (Figure 2; Figure 3). In both standard control MO-injected embryos and *ntn1a* 5-mispair control MO-injected embryos (Table 1), VaP and CaP developed normally. Thus, VaP axons remained stalled at the muscle pioneers in nearly every case and CaP axons extended to ventral muscle (Figure 2A; Figure 3). In *ntn1a* MO-injected embryos, both CaP and VaP axons extended out of the spinal cord along their normal path to the muscle pioneers (Figure 2B-B”), suggesting that as in other species [18], [19], [20], [21], zebrafish Ntn1a is not required for ventrally-projecting motor axons to exit the spinal cord. We followed development of five CaP and VaP pairs in *ntn1a* MO-injected embryos (Figure 2B; Figure 3). In these experiments, as in the controls, all labeled CaPs extended normal axons. In contrast, VaPs developed abnormally following Ntn1a knockdown. Instead of having axons that stalled at the muscle pioneers, as in the controls, all labeled VaPs in *ntn1a* MO-injected embryos had axons that extended beyond the muscle pioneers (Figure 2B). These results are consistent with our hypothesis that Ntn1a is a muscle pioneer-derived signal that breaks the equivalence between CaP and VaP by preventing one CaP/VaP axon from extending beyond the muscle pioneer intermediate target.

**Table 1:** MO sequences used to knock down Netrins, Dcc and Dscam and RT primer sequences used to confirm MO efficacy.

MO	MO Sequence (5′-3′)	RT-PCR primers (5′-3′)	Reference
*ntn1a* SBMO	ATGATGGACTTACCGACACATTCGT	*ntn1a* pre-mRNA (F1 X R1)*ntn1a* mRNA (F1 X R2)F1: CTTTCGGAGACGAAAACGAGR1: GTAGGCGCTTTCCAGAGATGR2: CTTTGCAGTAGTGGCAGTGG	Suli et al. 2007 [17]; Suli 2006 [26]
*ntn1a* TBMO	CGCCTTCCTCAGCCTCTCCTGTGCT	n/a	n/a
*ntn1a* 5-mispair MO	ATcATGcACTTAgCGACAgATTgGT (lower case letters are mismatches)	n/a	n/a
*ntn1b* SBMO	TAGTTTAGAAATGACTCACCGACAC	*ntn1b* pre-mRNA (F1 X R1)*ntn1b* mRNA (F1 X R2)F1: CCGACATCAAAGTGACCTTCR1: GAGCCATCCACACTTGTTGAR2: TGCACGTCGGTGTGATATAG	complementary to *ntn1b* exon 1/ intron 1–2 boundary
*ntn2* SBMO	TTTCGTGACTTACGTAAGCACTCGT	net2E1F: TCCGGAGTGTGATCGATGTAnet2E3R: CCTTTAGCACAGCGGTTACA	netrin2 SBMO3, Suli et al., 2007 [17]
*dcc* TBMO	GAATATCTCCAGTGACGCAGCCCAT	n/a	Suli et al., 2006 [26]
*dscam* SBMO	AAAGATCCTGAAATGCTCACCGGCC	*dscam* pre-mRNA (e2F X i2R)*dscam* mRNA (e2F X e3R)e2F: TTCTCAGTGAAGACCTACATTCCi2R: ATTCTGGGTAAGAGCTGTGAe3R: AATGCACTTGAAGACCGCTA	complementary to *dscam* exon2/intron 2–3 boundary
*dscam* TBMO	CGCTCCTTTCAATCTCCAAACTAAG	n/a	DS2M, Yimlamai et al., 2005 [25]
standard control TBMO	CCTCTTACCTCAGTTACAATTTATA	n/a	Gene Toolshttps://store2.gene-tools.com/node/7

Surprisingly, our time-lapse observations showed that following Ntn1a knockdown, CaP or VaP identity can be plastic and change over time. For example, in the pair of cells shown in Figure 2B, at 25 hours postfertilization (hpf), the axon of the green cell was stalled at the horizontal myoseptum, defining it as VaP, and the axon of the red cell extended into ventral muscle, defining it as CaP (Figure 2B'). However, by 6 hours later, the CaP axon had retracted to the horizontal myoseptum, thus the cell that was initially CaP had become VaP. In addition, the VaP axon had extended into ventral muscle, thus the cell that was initially VaP became CaP (Figure 2B”). We observed this dynamic change between CaP and VaP identity in two of the five labeled CaP and VaP pairs. Changes in CaP and VaP identity were not seen in time-lapse observations of many labeled CaP and VaPs under normal conditions [6], [7], nor in any of the eight labeled CaP and VaP pairs in standard control or *ntn1a* 5-mispair MO-injected embryos. These results are also consistent with our hypothesis that Ntn1a is a muscle pioneer-derived signal that breaks the equivalence between CaP and VaP and further suggest that when this signal is absent or reduced, the identity of these cells remains labile.

In addition to extending axons beyond the muscle pioneers, at 24 hpf, four of the five labeled VaPs in *ntn1a* MO-injected embryos had axons that extended branches along, but not beyond the horizontal myoseptum (Figure 2B). At 36 hpf, all four labeled VaPs retained this ectopic branch (Figure 2B). Previous studies showed that CaPs often extend transient branches in this region [6], [22], [23], but such branches have not previously been described for VaPs. These results suggest that, in addition to breaking the equivalence between CaP and VaP, Ntn1a restricts VaP axon branching, a function of Ntn-1 that has previously been described for retinal ganglion cells in mouse [24]. This additional function may be another manifestation of Ntn1a preventing VaP from extending an axon beyond the muscle pioneers or may reflect the involvement of two independent Ntn1a-mediated pathways in VaP development.

### Differential Netrin receptor expression does not distinguish CaP and VaP

**Figure 4:**
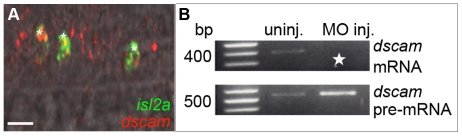
*dscam* mRNA is expressed in CaPs and VaPs and wildtype transcripts were knocked down with SBMOs. **A.** A projected confocal stack of *dscam* mRNA (red) and *isl2a* mRNA (a CaP/VaP motoneuron marker [41]) expression (green), reveals that at 24 hpf *dscam* is expressed in both CaP and VaP (*; CaP and VaP pair to left, single CaP to right) as well as other cells that are likely motoneurons. **B.** RT-PCR results confirming uninjected embryos have wildtype *dscam* mRNA (395 bp band) while MO-injected embryos have increased *dscam* pre-mRNA (490 bp band). Starred lanes show *dscam* transcripts are absent after injecting 8.4 ng of *dscam* SBMO. **Scale bar**  = 20 µm.

**Figure 5:**
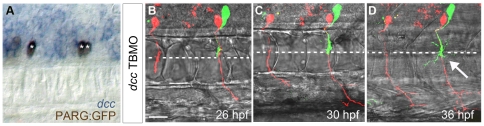
Dcc is necessary to prevent a second CaP/VaP axon from extending beyond the muscle pioneers. **A**. Brightfield image of lateral view of 24 hpf embryo focusing on the spinal cord. *dcc* mRNA (blue) is co-localized with a motoneuron marker, PARG:GFP (brown; see also [47]). The left segment shows one CaP (*) expressing *dcc*. The right segment shows two CaP/VaPs (**) expressing *dcc*. *dcc* is also expressed in additional cells that are likely other motoneurons and neighboring interneurons. **B–D.** Projected confocal stacks of living dye-labeled motoneurons in the same *dcc* TBMO-injected embryo at three developmental stages. Punctate red and green fluorescence within the spinal cord but not in CaP or VaP in this and other figures represents dye leakage from the labeling procedure. The left segment shows a CaP (red) in a segment lacking VaP; the right segment shows both CaP (red) and VaP (green). **B.** At 26 hpf the VaP axon does not extend beyond the level of the muscle pioneers (dashed line) but CaP axons extend farther. **C.** By 30 hpf, CaP axons have extended farther ventrally but the VaP axon still remains near the muscle pioneers. **D.** By 36 hpf, CaP axons have reached the ventral aspect of the myotome, providing evidence that Dcc is unnecessary for CaP axon extension. The VaP axon has extended branches beyond and along the muscle pioneers (arrow). **Scale bar**  = 20 µm.

Because different combinations of Netrin receptors can lead to different responses to Netrin, we asked whether CaP and VaP were distinguished by their expression of Netrin receptors. We analyzed expression of several Netrin receptor genes, including *dcc*, *dscam*, *neo1*, *neo2*, and *unc5b* (see also Materials and Methods), that mediate either attraction or repulsion to Netrin [12], [14]. Of these genes we found that only *dscam* and *dcc* are expressed in motoneurons. Moreover, *dscam* (Figure 4A) and *dcc* (Figure 5A) are expressed in both CaP and VaP. These results refute the simple hypothesis that differences in Netrin-receptor gene expression between CaP and VaP result in the differential responses of their axons to Ntn1a.

### Dcc is a Netrin receptor necessary for VaP fate

We tested whether Dscam is necessary for VaP axons to stop at the horizontal myoseptum by knocking down Dscam with a previously published translation blocking (TB) MO [25] and a SBMO (Figure 4B; Table 1). Embryos injected with *dscam* MO exhibited a range of previously described phenotypes associated with defects in gastrulation that affect mesoderm and neural plate formation (data not shown; [25]). These early defects precluded using MOs to investigate whether Dscam is necessary for VaP cell fate.

To test whether Dcc is required for VaP axons to stop at the horizontal myoseptum, we knocked down Dcc using a previously validated TBMO [17], [26]. We followed five dye-labeled CaP and VaP pairs in *dcc* MO-injected embryos (Figure 5B–D; Figure 3). As for Ntn1a morpholino knockdown, CaP axon extension was not altered by Dcc morpholino knockdown. In contrast, following Dcc knockdown, VaPs extended axons beyond the muscle pioneers (Figure 5B–D). Initially all of the labeled VaP axons paused at the muscle pioneers in *dcc* MO-injected embryos (Figure 5B), but at later stages, the majority of labeled VaP axons had extended beyond the muscle pioneers (Figure 5D; Figure 3). Thus, following Dcc knockdown, VaPs failed to adopt their normal fate and instead extended axons beyond the muscle pioneers. These results suggest that Dcc mediates the ability of VaP to respond to Ntn1a. We cannot rule out the possibility that this is due to autonomous effects of Dcc in both CaP and VaP.

### Additional Netrins act as a failsafe mechanism to prevent VaP from becoming a second CaP

In *ntn1a* MO-injected embryos and in *dcc* MO-injected embryos, VaPs extended axons beyond the muscle pioneers and thus resembled CaPs. However, these cells differed from normal CaPs because their axons remained shorter; we refer to cells with intermediate axon lengths as “CaP-like” (Figure 2B; Figure 5D). To learn more about what prevented these CaP-like cells from fully transforming into CaPs, we examined the regions where their axons stopped.

**Figure 6:**
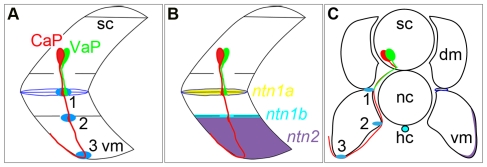
Netrin mRNAs are expressed at intermediate targets (blue ellipses) of the CaP axon as it extends ventrally. **A.** Schematized view of one spinal hemisegment including spinal cord (sc) and overlying muscle. CaP (red) and VaP (green) axons contact muscle pioneers (blue) at the first intermediate target (**1**). Later the CaP axon contacts the second intermediate target (**2**) at the level of the hypochord (hc), immediately below the notochord (nc) and extends farther ventrally to a third intermediate target (**3**) in ventral myotome (vm). **B.** Schematized view detailing expression of *ntn1a* mRNA (yellow) in the muscle pioneers, *ntn1b* mRNA (light blue) near the hypochord, and *ntn2* mRNA (purple) in ventral myotome. **C.** Schematized view of a transverse section through an embryonic zebrafish trunk showing CaP and VaP cell bodies in the spinal cord, intermediate targets of their ventrally-projecting axons, and expression of *netrin* mRNAs in intermediate targets.

Following Ntn1a or Dcc knockdown, CaP-like axons did not progress beyond the level of the hypochord, an embryonic structure located along the length of the ventral aspect of the notochord (http://zfin.org/action/anatomy/term-detail?anatomyItem.zdbID=ZDB-TERM-100331-29) (Figure 6; Figure 3). This region is adjacent to the second intermediate target where CaP axons normally pause during extension toward ventral muscle [27]. The most ventral aspect of the myotome defines a third intermediate target where CaP axons normally pause during their extension [27]. However, we never saw CaP-like axons progress to the third intermediate target following knockdown of Ntn1a or Dcc.

**Figure 7:**
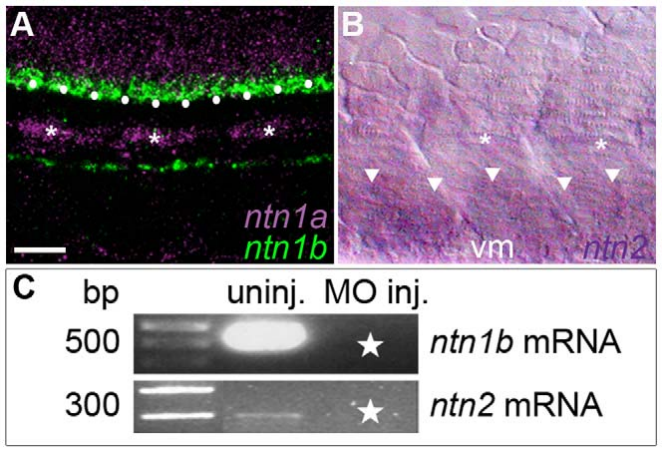
Netrin mRNAs are expressed in intermediate targets of the CaP axon and wildtype transcripts were knocked down with SBMOs. **A**. Projected confocal stack of *ntn1a* and *ntn1b* mRNA expression in zebrafish trunk at 24 hpf; *ntn1a* is expressed in spinal cord (purple) and muscle pioneers (purple and *). *ntn1a* staining was false colored purple. *ntn1b* mRNA (green) is expressed in floor plate (white dots) and hypochord, located ventrally to the notochord. **B**. Brightfield image of lateral surface of zebrafish trunk at 24 hpf reveals *ntn2* is expressed in ventral somite (white arrowheads). Asterisks denote muscle pioneers, cells that do not express *ntn2*. C. RT-PCR results confirming uninjected embryos have wildtype *ntn1b* (500 bp band) and *ntn2* (300 bp band) transcript. Starred lanes show *netrin* transcripts are reduced or absent after injecting 10.0 ng of *ntn1b* SBMO or 5.0 ng of *ntn2* SBMO. Multiple samples were run in the gels for *ntn1b* and *ntn2*. Lanes containing samples unrelated to the *netrins* were cropped from the figure. **Scale bar**  = 20 µm.

All three CaP intermediate target regions express *netrin* mRNAs. As described above, the first intermediate target, the muscle pioneers, expresses *ntn1a* (Figure 1A; Figure 6B, C; Figure 7A). The hypochord at the level of the second intermediate target expresses *ntn1b* during the time that the CaP axon is navigating along this region of its pathway (Figure 6B, C; Figure 7A) [28], [29]. Ventrolateral myotome cells in the region of the third intermediate target express *netrin 2* (Figure 6B,C; Figure 7B) [28]. The cells expressing *ntn1b* and *ntn2* are at different mediolateral positions within the embryo (Figure 6C). The *ntn1b*-expressing hypochord is located along the axial midline. In contrast, the *ntn2*-expressing cells are located ventrolaterally within the somite.

**Figure 8:**
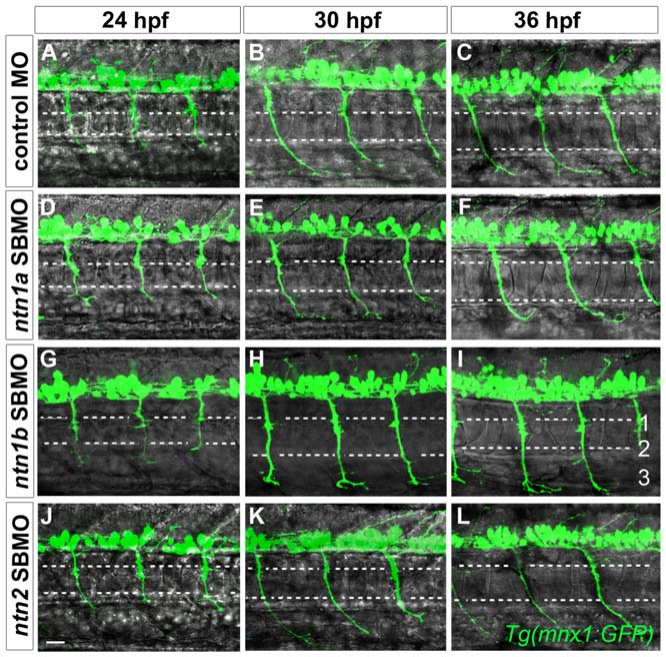
Netrins are unnecessary for CaP axons to extend to intermediate targets. Projected confocal stacks of GFP-expressing motor axons in living *Tg*(*mnx1:GFP*) embryos injected with various MOs. **A–C.** In standard control MO-injected embryos CaP axons extend to the second intermediate target by 24 hpf (**A**), the third intermediate target by 30 hpf (**B**), and by 36 hpf have extended past the third intermediate target and wrap around the ventral aspect of the myotome (**C**). In *ntn1a* SBMO (**D–F**), *ntn1b* SBMO (**G–I**), and *ntn2* SBMO (**J–L**) injected embryos the time course of CaP axon outgrowth was the same as in controls (**A–C**). **Scale bar**  = 20 µm.

**Figure 9:**
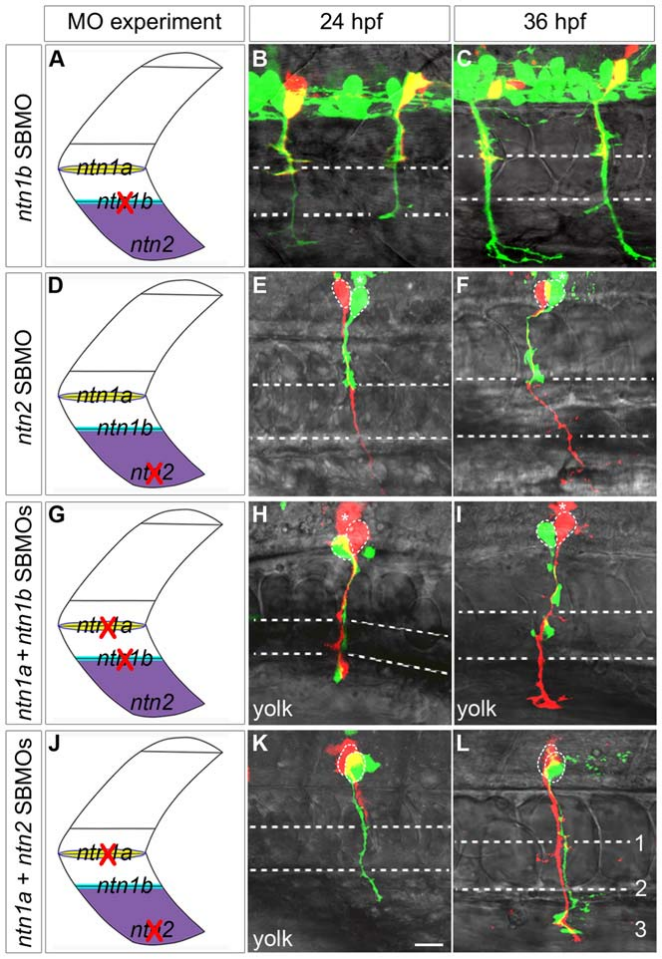
Netrins can prevent extension of a second CaP axon into ventral muscle. Projected confocal stacks of living dye-labeled motoneurons in embryos injected with various combinations of MOs. **A, D, G, J**. Cartoons of a trunk spinal hemisegment detailing *ntn1a*, *ntn1b*, and *ntn2* mRNA expression. The red X indicates which *netrin* transcript was knocked down in adjacent panels. **B–C**. *ntn1b* SBMO-injected *Tg*(*mnx1:GFP*) embryo showing motoneurons (green) and rhodamine dextran-labeled VaPs (yellow). At 24 (**B**) and 36 hpf (**C**) VaP axons are stopped at muscle pioneers (upper dashed line). **E, F, H, I, K, L**. Embryos with individually-labeled CaP and VaP pairs. Dashed ellipses outline motoneuron cell bodies. In some cases interneurons (*) were also injected with dye during the labeling procedure. **E–F**. In *ntn2* SBMO-injected embryos the VaP axon (green) stalled at the muscle pioneers while the CaP axon (red) extended into ventral muscle at 24 hpf (**E**) and at 36 hpf (**F**). **H–I**. In *ntn1a* plus *ntn1b* SBMO-injected embryos two axons extended into ventral muscle at 24 hpf (**H**), but by 36 hpf (**I**) one axon retracted to the level of the second intermediate target (lower dashed line), becoming CaP-like (green). **K–L**. In the absence of *ntn1a* and *ntn2* only one axon (green) initially extended to the third intermediate target (**K**). However, by 36 hpf a second axon (red) extended to the third intermediate target, thus both cells become CaPs (**L**). Dashed ellipses in **L** outline the motoneuron cell bodies. **Scale bar**  = 20 µm.

Because multiple Netrins act in concert during development to guide the axons of other spinal neuron populations to their targets [30], we asked whether combinations of Netrins are required for the CaP axon to leave the spinal cord and whether Netrins expressed by CaP intermediate targets are necessary for normal CaP axon extension. To address these questions, we knocked down the individual Netrins using MOs (Figure 7C) and followed CaP development over time. We found that CaPs extended normally following knockdown of each of the Netrins individually (Figure 8; see also Figure 2 & Figure 9) as well as following knockdown of pairs of Netrins (Figure 9). Simultaneous injection of MOs to all three Netrins produced widespread defects and neuronal cell death (data not shown) that prevented us from examining motoneuron development in these embryos. Our morpholino knockdown results suggest that neither individual Netrins nor pairs of Netrins act to guide CaP axons out of the spinal cord or to their intermediate targets. However, in the absence of knowledge of Netrin protein distribution, we cannot exclude the possibility that Ntn1b and/or Ntn2 function redundantly to guide CaP axons from afar.

Similar to our results with CaP, our morpholino knockdown experiments suggested that Ntn1a is unnecessary for VaP axons to extend out of the spinal cord and to navigate to the first intermediate target. VaP axons do not encounter the second and third intermediate targets and thus are unlikely to be affected by either *ntn1b* or *ntn2* during normal development. To test this hypothesis, we knocked down Ntn1b and Ntn2 individually. We used a splice-blocking MO to knock down Ntn1b and followed seven dye-labeled CaP and VaP pairs. Five of seven labeled VaPs did not extend axons beyond the muscle pioneers (Figure 9A–C; Figure 3). We knocked down Ntn2 with a splice-blocking MO and followed four dye-labeled CaP and VaP pairs. Axons of all four VaPs stalled at the muscle pioneers (Figure 9D–F; Figure 3). These results suggest that neither Ntn1b nor Ntn2 alone is necessary for the VaP axon to stop at the horizontal myoseptum, consistent with our hypothesis that VaP axons do not respond to these Netrins during normal development.

Because CaP-like axons stopped at the second intermediate target following knockdown of *ntn1a* or *dcc*, we hypothesized that *ntn1b* and/or *ntn2* might affect CaP-like axons similarly to the way *ntn1a* affects VaP axons. We therefore asked whether, in the absence of a Netrin signal from the muscle pioneers, Ntn1b and/or Ntn2 could prevent a second CaP axon from extending toward more distal intermediate targets, causing the cell to become CaP-like. To test this hypothesis, we injected embryos either with *ntn1a* plus *ntn1b* MOs or with *ntn1a* plus *ntn2* MOs, followed development of dye-labeled CaP and VaP pairs, and obtained the following results for the two different pairs of MO combinations.

#### Ntn1a plus Ntn1b knockdown

We followed seven dye-labeled pairs of CaPs and VaPs in *ntn1a* plus *ntn1b* MO-injected embryos (Figure 9G–I; Figure 3). All seven labeled VaP axons extended beyond the horizontal myoseptum and stopped at the second intermediate target, becoming CaP-like. Interestingly, during outgrowth one of these cells initially extended an axon beyond the second intermediate target, but then retracted that axon back to the second intermediate target by 36 hpf.

#### Ntn1a plus Ntn2 knockdown

We followed twelve dye-labeled pairs of CaPs and VaPs in *ntn1a* plus *ntn2* MO-injected embryos (Figure 9J-L; Figure 3). All twelve labeled VaP axons extended beyond the horizontal myoseptum. Eight of these cells became CaP-like, with axons that stopped at the second intermediate target. Four of these cells extended axons that reached the third intermediate target and in all four cases both labeled cells resembled normal CaPs.

During normal development, a Netrin signal from the first intermediate target breaks the equivalence between CaP and VaP by preventing one of the CaP/VaP axons from extending farther ventrally. The VaP axon does not normally encounter the subsequent intermediate targets. Our results demonstrate that under experimental conditions that allow both CaP/VaP axons to extend beyond the first intermediate target, one of the axons is prevented from extending farther by a Netrin signal from a subsequent intermediate target. Together these results suggest that the same set of signaling interactions can occur at multiple locations, providing a failsafe mechanism for preventing formation of a second CaP, and revealing a new role for intermediate targets in breaking neuronal equivalence.

### Motoneurons are unnecessary for ntn1a expression by the muscle pioneers

**Figure 10:**
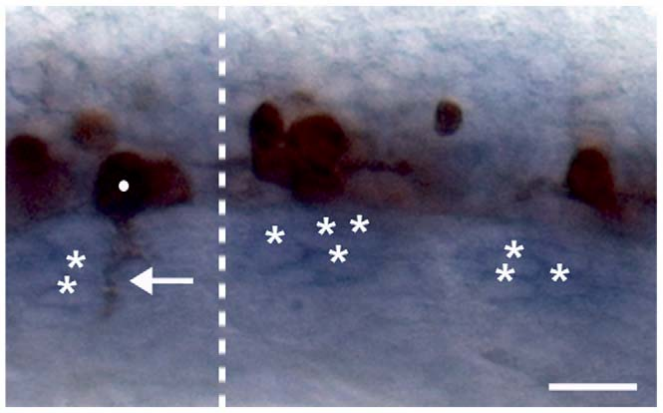
A CaP/VaP-derived signal is not required for expression of the muscle pioneer-derived Netrin signal. This image shows an 18 hpf embryo from which CaP/VaPs were removed before axogenesis from the two segments right of the dotted line, whereas CaP/VaPs were not removed from the segment left of the dotted line. The zn1 and znp1 antibodies used to label the motoneuron axons recognize both motoneuron somata and the somata of some other neurons in the ventral spinal cord [66], [71]. The segment on the left shows a CaP/VaP soma (dot) and its axon (arrow) labeled by the antibodies. The two segments on the right show somata of other neurons, but no CaP/VaP somata or motor axons. *ntn1a* mRNA (blue) was expressed in the muscle pioneers (*) whether CaP/VaPs were present (left) or absent (right). **Scale bar**  = 20 µm.

We previously proposed that a CaP-derived signal was responsible for the ability of the muscle pioneers to prevent the VaP axon from extending farther ventrally [6]. Because Ntn1a is necessary for the VaP axon to remain stalled at the muscle pioneers, our previous model raises the possibility that contact with a CaP/VaP axon is required for the muscle pioneers to express *ntn1a* mRNA. To test this possibility, we manually removed CaP/VaPs prior to axon outgrowth as described in Appel et al. [69]. Contrary to our expectation, *ntn1a* expression in the muscle pioneers was not altered by the absence of CaP/VaPs (Figure 10). This result shows that *ntn1a* mRNA expression in the muscle pioneers is independent of CaP/VaP axon contact. However, localization of Netrin protein is highly regulated [11] and may not parallel mRNA expression. Thus it remains possible that CaP/VaP contact alters Ntn1a protein distribution on the muscle pioneers. Because there are currently no commercially-available antibodies that recognize zebrafish Ntn1a, and we have not had success generating antibodies that recognize zebrafish Ntn1a, we were unable test this hypothesis.

## Discussion

Our data suggest a novel function for intermediate targets in diversifying a neuronal population. We show that in zebrafish, an intermediate target-derived signal causes a pair of developmentally equivalent motoneurons to adopt distinct fates by preventing the axon of one of them from extending beyond that intermediate target. During neuronal differentiation, the axons of many neurons encounter intermediate targets that direct their subsequent development [31], [32], [33], [34], in many cases by altering gene expression within the neuron [35], [36], [37]. Intermediate targets are well known as locations where axons make pathway choices that lead to correct circuit formation [32], [33], [34]. Our results suggest that at least some intermediate targets may also affect formation of neuronal circuitry by influencing cell fate specification.

### CaP and VaP respond differently to intermediate target-derived Netrins

Our morpholino knockdown experiments suggest that Netrins are unnecessary for CaP or VaP to extend axons to their targets, consistent with what has been described from other knockdown studies of spinal motoneurons in zebrafish and chick and mutant studies in mouse [18], [20], [21], [38], [39], [40]. However, after they have paused at the first intermediate target, VaP axons respond very differently to Netrin than do CaP axons. One interpretation of this difference in response to Netrin is that CaP and VaP are intrinsically different, and thus have distinct fates before their axons encounter the first intermediate target. An intrinsic difference in the neurons may be reflected in differences in Netrin receptor distribution or some yet to be described gene(s). However, the idea that differential Netrin receptor distribution on CaP and VaP distinguishes the cells is not supported by data presented here showing that both CaP and VaP express RNA transcripts for the same Netrin receptors. Nor is it consistent with expression data of many other genes, none of which are differentially expressed in CaP and VaP [41], [42], [43], [44], [45], [46], [47], [48]. Moreover, our previous cell ablation and transplantation experiments showed that interactions among the axons of the two CaP/VaPs and the MPs are the crucial step in determining which cell becomes VaP [6]. Thus, even if CaP and VaP are distinct in some way before their axons encounter the muscle pioneers, this distinction can be overridden by interactions among the cells, showing that CaP and VaP remain developmentally equivalent. These data provide strong support for the idea that signaling at the muscle pioneers, rather than intrinsic differences between CaP/VaPs, determines which cell adopts the VaP fate.

We still do not understand how the two CaP/VaPs respond differently to Netrin. We previously proposed that the first CaP/VaP to contact the muscle pioneers invariably becomes CaP [6]. If this is the case, then Netrin signaling through Dcc in CaP could promote signaling to the second CaP/VaP, forcing it to become VaP. We attempted to test this proposal in two ways. We labeled individual CaP/VaPs with different fluorescent dyes prior to axogenesis and imaged them continuously as their growth cones extended toward the muscle pioneers. However, the prolonged illumination necessary to follow these cells over the relevant time caused them to die, confirming previous results that demonstrated labeled cells are very sensitive to photodamage [9]. We also imaged axons of pairs of CaP/VaPs as they extended to muscle pioneers in transgenic lines expressing GFP in motoneurons. However, because the axons are closely apposed, we could not determine which cell's axon contacted the muscle pioneers first. Thus, we were unable to determine whether the first CaP/VaP that contacts the muscle pioneers invariably becomes CaP. Although, both cells express the same Netrin receptor mRNAs, that does not necessarily mean that the receptors act equivalently in CaP and VaP. Dcc and other Netrin receptors can be modified posttranslationally and can activate a variety of downstream effectors that in turn regulate receptor levels at the plasma membrane [39], [49], [50], [51], [52]. Thus we propose that differential receptor activity underlies differences in the responses of CaP and VaP to Netrins. Because we do not yet have an indication of how receptor activity might differ between these two cells, this hypothesis awaits future testing.

### Ntn1a knockdown reveals possible roles for other intermediate target-derived Netrins

We previously showed that two CaP/VaPs and the muscle pioneers are required for one of the CaP/VaPs to adopt the VaP fate. Here we provide evidence that Ntn1a is a muscle pioneer-derived signal involved in this process. Following knockdown of Ntn1a, the two CaP/VaPs remain equivalent. Surprisingly, we also found that following knockdown of Ntn1a, another Netrin, produced by a more distal intermediate target, can interact with the axons of the CaP/VaPs to break the equivalence of the two cells later in their development. Although this particular pair of motoneurons would not normally interact with intermediate targets distal to the muscle pioneers, such an interaction may be important for determining the fates of later-developing zebrafish motoneurons that also extend axons to the same intermediate targets [27].

Previous studies have reported motor axon guidance defects in mutants or following knockdown of potential motor axon guidance cues expressed by the muscle pioneers or their precursors [53], [54], [55]. In each of these cases, ventrally-projecting motoneurons were assayed by labeling with antibodies, thus CaP and VaP axons, which are closely apposed to one another, could not be distinguished. Because CaP and VaP were not differentially labeled in these studies, it is not possible to conclude whether any of these molecules affects these two motoneurons differentially. It would be interesting to investigate these guidance cues in more detail in the future, as it could provide insight into the complex signaling interactions that may occur at intermediate targets to specify cell fate.

### Do other intermediate targets play a role in breaking neuronal equivalence?

The type of mechanism we have described here could also be important for breaking the equivalence between neurons in other situations. For example, during development of neuromuscular connectivity in many vertebrates, interactions among motoneurons that belong to a specific motor pool, and fibers within the muscle these motoneurons innervate, sculpt specific circuits to refine initially diffuse and redundant synaptic connections [56]. Netrin is expressed in developing muscle in avian embryos [38], although it is not required for motor axon extension extension [20], [21]. Whether it is required to diversify motoneurons during formation of specific circuits has not been addressed in avian or mammalian models. Similarly, neurons within some brain regions establish topographic maps by making connections with distinct synaptic partners (reviewed by [32], [57]). A common theme in each of these cases is that neurons that are initially equivalent later develop distinct synaptic connections. Many mechanisms have been described that lead to this outcome (reviewed by [32], [57]). However, one mechanism that has not been previously discussed is that signaling interactions among equivalent neurons and intermediate targets could break the equivalence between the neurons, similar to the way that muscle pioneer-derived Ntn1a breaks the equivalence between two CaP/VaPs. Such a mechanism would ensure that initially equivalent neurons followed divergent developmental pathways only if they encountered an appropriate signal from an intermediate target. In the absence of that signal, the neurons would remain equivalent and able to follow the same developmental pathway, perhaps making a later fate decision if an encounter with a more distal intermediate target broke their equivalence.

The mechanism we describe in this paper suggests a previously unrecognized role for intermediate targets, not just as guideposts for directing axon pathway navigation, but also as signaling centers that affect cell fate. It will be interesting to learn whether this mechanism is widespread during development.

## Materials and Methods

### Ethics statement

The protocol for this study was reviewed and approved by the University of Oregon Institutional Animal Care and Use Committee (08-21RR).

### Animal husbandry and lines

Zebrafish embryos were obtained from natural spawning of ABC wild types, or *parg^mn2Et^* [42] or *Tg*(*mnx1:GFP*)*ml2* [58] transgenic lines. Fish were staged by hours postfertilization at 28.5°C (hpf) [59].

### RNA in situ hybridization

RNA in situ hybridization using antisense-digoxigenin labeled *deleted in colorectal carcinoma* (*dcc*, ZFIN ID: ZDB-GENE-011101-2) [60], *neogenin 1* (*neo1*, ZFIN ID: ZDB-GENE-021031-1) [61], *neogenin 2* (*neo2*, GenBank Acc: AL907501), and *uncoordinated 5b* (*unc5b*, ZFIN ID: ZDB-GENE-041213-1) [62] oligonucleotide probes was carried out according to previously described protocols [63]. Fluorescent in situ hybridization using *Down syndrome cell adhesion molecule* (*dscam*, ZFIN ID: ZDB-GENE-050310-7) [25], *islet 2a* (*isl2a,* ZFIN ID: ZDB-GENE-980526-562; a CaP/VaP marker; [41]), *netrin 1a* (*ntn1a*, ZFIN ID: ZDB-GENE-990415-169) [16], *netrin 1b* (*ntn1b*, ZFIN ID: ZDB-GENE-990415-168) [29], *netrin 2* (*ntn2*, ZFIN ID: ZDB-GENE-050310-2) [28], antisense probes was performed as previously described [64] with the following modifications [65]: probes against *ntn1a* mRNA and *isl2a* mRNA were made with dinitrophenyl (DNP) labeled nucleotides (PerkinElmer Life and Analytical Sciences, Inc., Shelton, CT, USA). Anti-DNP-HRP conjugated antibody and subsequent development with the TSA/Cy5 fluorescent system (PerkinElmer Life and Analytical Sciences, Inc., Shelton, CT, USA) was used to visualize mRNA expression.

### Immunohistochemistry

The following primary antibodies (Abs) were used: monoclonal mouse zn-1 [66], monoclonal mouse znp-1 [66], monoclonal mouse F59 [67], and JL-8 monoclonal mouse anti-GFP (Clontech Laboratories, Inc., Mountain View, CA, USA). The following secondary antibodies were used: goat anti-mouse Alexa Fluor® 488 (Invitrogen-Molecular Probes; Eugene, OR, USA), goat anti-mouse Alexa Fluor® 633 (Invitrogen-Molecular Probes; Eugene, OR, USA), and goat anti-mouse-HRP (The Jackson Laboratory, Bar Harbor, ME, USA).

Motoneuron cell bodies and axons were visualized with combined antibody labeling using zn1 and znp1, referred to as zn1/znp1 (1∶50/1∶1000) [45], or with transgenic lines that express GFP in motoneurons, *Tg*(*mnx1:GFP*)*ml2* or *parg^mn2Et^* [47]. Slow muscle fibers, including muscle pioneers, were visualized by F59 antibody staining (1∶10) using the antibody protocol described by Hutchinson et al. [45]. Anti-GFP antibody staining was used to visualize GFP expression in embryos that were first taken through the RNA in situ hybridization (ISH) protocol described above [63]. Briefly, embryos were fixed after ISH for 1 hour in 4% PFA in 1x PBS at 4°C, washed for 30 minutes in PBST, and incubated with primary antibody, JL-8 anti-GFP (1∶200). Then embryos were incubated with either of the following secondary antibodies: goat anti-mouse-HRP (1∶200) or goat anti-mouse Alexa Fluor® 633 (1∶750), as described in Hutchinson et al. [45]. GFP expressing cells in embryos incubated with the HRP-conjugated secondary were visualized by diaminobenzidine (DAB) development as described in Appel and Eisen [63].

### Morpholinos

#### Morpholino sequences and reverse transcription PCR

The MOs described in Table 1 were used to knock down zebrafish Netrins, Dcc, and Dscam or as controls to confirm MO specificity. All MOs were designed by Gene Tools (Philomath, OR, USA). Reverse transcription (RT)-PCR was used to assay wild-type transcript knockdown in embryos injected with splice-blocking MOs. Table 1 lists primer pairs used to determine efficacy of MO knockdown. PCR conditions for *netrin* transcripts were as described in Suli et al. [26]. Platinum PCR SuperMix (Invitrogen; Carlsbad, CA, USA) was used to amplify *dscam* transcripts. PCR conditions for *dscam* were as follows: 5 min 94°C; followed by 30 cycles of 94°C, 30 sec/55°C, 30 sec/68°C, 2 min; followed by 5 min 68°C.

#### Morpholino injections

The following amounts of MO diluted in 0.2% phenol red solution in water were injected into 1–2 cell stage embryos: standard control MO, 0.5–5.3 ng; *ntn1a* SBMO, 2.1–8.9 ng; *ntn1b* SBMO, 5.6–15 ng; *ntn2* SBMO, 5.2–18 ng; *dcc* TBMO, 0.5–5.2 ng; *dscam* SBMO, 4.2–8.4 ng; *dscam* TBMO, 4.5–15.3 ng. For double MO injections the same amount of MO was injected as individual MO experiments, but in combination. Injected amounts were calculated by measuring the diameter of a MO bolus injected into an oil droplet.

### Single cell labeling

Individual primary motoneurons were labeled as previously described [6], [68] with the following modification: cells were injected with a 5% or 2.5% solution of either tetramethylrhodamine-dextran or Alexa Fluor®488-dextran (3000 MW, anionic; Invitrogen-Molecular Probes, Eugene, OR, USA) in 0.2M KCl. To avoid photodamaging dye-injected cells, they were allowed to recover for one hour in a dark, 28.5°C incubator before imaging, were imaged at only three time points over 12 hours, and were scanned for less than five minutes. Some labeled cells did not survive. Since previous studies showed that VaP can become CaP even when CaP is ablated relatively late [6], we only included CaP and VaP pairs in which both cells survived to 36 hpf, the end point of our observations.

### Motoneuron removal experiments

Motoneurons were removed as previously described [69]. Briefly, embryos were mounted in agar, a small hole dissected in the skin, and individual motoneurons removed by gentle suction from a micropipette.

### Microscopy

Images of fixed zebrafish embryos were captured on a Zeiss Axioplan equipped with a digital camera, or on a Zeiss Pascal confocal microscope. Images of living embryos were captured at 24, 30 and 36 hpf on a Zeiss Pascal confocal microscope using a 40x water immersion objective. The brightness and contrast of images was adjusted with Zeiss LSM Image Browser (Version 4.2.0.121, Carl Zeiss MicroImaging, Thornwood, NY, USA), or Photoshop CS4 Extended (Version 11.0, Adobe Systems, Inc., San Jose, CA, USA).
